# Strategies supporting the prevention and control of neglected tropical diseases during and beyond the COVID-19 pandemic

**DOI:** 10.1186/s40249-020-00701-7

**Published:** 2020-07-10

**Authors:** John P. Ehrenberg, Xiao-Nong Zhou, Gilberto Fontes, Eliana M. M. Rocha, Marcel Tanner, Jürg Utzinger

**Affiliations:** 1 Avenida Cedro 9, # 303, Cholul, Merida, Yucatan Mexico; 2 Formerly World Health Organization, Regional Office for the Western Pacific, Manila, The Philippines; 3 National Institute of Parasitic Diseases at the Chinese Center for Disease Control and Prevention & Chinese Center for Tropical Diseases Research, Shanghai, People’s Republic of China; 4 School of Global Health, Chinese Center for Tropical Diseases Research - Shanghai Jiao Tong University School of Medicine, Shanghai, People’s Republic of China; 5 Laboratory of Parasitology, Central-West Campus, Federal University of São João del Rei, Divinopolis, Minas Gerais Brazil; 6 Swiss Tropical and Public Health Institute, Basel, Switzerland; 7 University of Basel, Basel, Switzerland

**Keywords:** COVID-19, Emerging and re-emerging diseases, Multi-sectorial approaches, Neglected tropical diseases, Zoonotic diseases

## Abstract

Emerging and re-emerging zoonotic diseases represent a public health challenge of international concern. They include a large group of neglected tropical diseases (NTDs), many of which are of zoonotic nature. Coronavirus disease 2019 (COVID-19), another emerging zoonotic disease, has just increased the stakes exponentially. Most NTDs are subject to the impact of some of the very same human-related activities triggering other emerging and re-emerging diseases, including COVID-19, severe acute respiratory syndrome (SARS), bird flu and swine flu. It is conceivable that COVID-19 will exacerbate the NTDs, as it will divert much needed financial and human resources. There is considerable concern that recent progress achieved with control and elimination efforts will be reverted. Future potential strategies will need to reconsider the determinants of health in NTDs in order to galvanize efforts and come up with a comprehensive, well defined programme that will set the stage for an effective multi-sectorial approach. In this Commentary, we propose areas of potential synergies between the COVID-19 pandemic control efforts, other health and non-health sector initiatives and NTD control and elimination programmes.

## Background

Humanity faces a series of public and global health issues. For instance, there is a wide range of emerging and re-emerging zoonotic diseases, which affect a large number of individuals worldwide [1]. Hence, these diseases should be an important component of the current public health agenda, as they represent a serious public health threat. Yet, this is not the case. Coronavirus disease 2019 (COVID-19), another emerging zoonosis [2], has just increased the stakes exponentially [3]. The emergence of severe acute respiratory syndrome coronavirus 2 (SARS-CoV-2), the causative agent of COVID-19, is thought to have originated from a bat reservoir. It is one of seven coronaviruses known to infect humans.

Emerging and re-emerging diseases share a number of determinant factors, one of which is particularly critical: the impact of human activities on the environment [4]. Anthropogenic climate change is the most prominent [5]. Yet, other determinants, such as armed conflicts and war, changing land-use patterns, migration, unprecedented levels of transferring people and goods, and social inequity are known to pose major threats to human health and well-being [6, 7]. The gap between the “haves” and “have-not” is increasing even within the new developing economies. Vulnerable populations throughout the world, such as ethnic minorities, not only have less access to health care but are also disproportionally affected by a wide range of neglected tropical diseases (NTDs) [8, 9]. COVID-19 is clearly illustrating the importance of tackling these determinants, as even the largest economies in the world, including the United States of America, the People’s Republic of China and countries in Europe are not spared the consequences.

NTDs comprise an important group of bacterial, parasitic and viral diseases, many of which are of zoonotic nature. Yet, as their name suggests, they continue to be neglected by most of the affected countries’ health agendas. Throughout the world, impoverished populations are heavily burdened with NTDs and often lack access to primary health services [10]. The NTDs that affect people living in vulnerable conditions create social and financial burdens, which threaten the individual and their communities [10]. In spite of notable progress in controlling or even eliminating some NTDs (e.g. dracunculiasis, human African trypanosomiasis, lymphatic filariasis and onchocerciasis), their importance in terms of disease burden remains considerable [11, 12]. Importantly though, NTDs remain for the most part underfunded and efforts to combat them rely on the good will of the private sector and philanthropy. For example, onchocerciasis elimination in the Americas relied on a successful partnership involving The Carter Center, Merck and endemic countries, among other partners, and the local communities [12]. COVID-19 threatens hard won gains in NTD control and elimination efforts.

We are just beginning to appreciate the enormous economic impact COVID-19 is having across the world. It is conceivable that the economic crisis will drag millions of yet more people into poverty. It is not hard to imagine what effect this will have on the large populations affected and at-risk of NTDs. Estimates of the combined burden of the very first list comprising only 13 NTDs, i.e. 56.6 million disability-adjusted life years (DALYs) exceeded that of malaria and tuberculosis and even approached that of human immunodeficiency virus/acquired immunodeficiency syndrome (HIV/AIDS) [13]. According to 2015, estimates of the population at-risk in the Western Pacific region (comprising 37 countries) stood at 200 million individuals for schistosomiasis alone [9]. All of these countries now face the imminent danger of the aftermath of COVID-19.

## What do COVID-19 and NTDs have in common?

COVID-19 and most NTDs are characterized by complex life cycles of the aetiological agents, which might involve one or more hosts as well as one or more species of vectors. Many NTDs are considered zoonosis because they affect primarily animal hosts but can jump species and affect humans, often with increased virulence and pathogenicity. This jump is almost always related to human activities and their impact on the environment. The list of NTDs is long [13, 14]. Out of the currently 20 NTDs listed by the World Health Organization (WHO) [15], this Commentary highlights four zoonotic NTDs, in order to illustrate prevention and control challenges, which are likely to increase in face of the unfolding COVID-19 pandemic [14, 16] (Table 1). The four selected NTDs cause considerable morbidity, might even lead to death and all have a significant economic impact on the already impoverished populations they affect. We believe that the economic impact of COVID-19 on these populations will be severe and long lasting.

**Table 1:** Similarities and differences between COVID-19 and four selected zoonotic neglected tropical diseases

General aspects	COVID-19	Schistosomiasis	Cystic echinococcosis	Leishmaniasis	Human African trypanosomiasis
**Aetiological agent**	SARS-CoV-2	*Schistosoma* spp.	*Echinococcus* spp.	*Leishmania* spp.	*Trypanosoma* spp.
**Transmission route**	• Animal-to-human • Human-to-human	• Human-to-snail-to-human (might involve another host, e.g. water buffalo)	• Dog-to-sheep-to-dog (humans as accidental or aberrant intermediate hosts) • Different animals act as either definitive or intermediate host	• Human-to-insect vector-to-human, other mammals (e.g. dogs) can act as host	• Human-to-insect vector-to-human, occasionally another mammal (e.g. wild ungulates) acts as reservoirs
**Prevention**	• Avoid close contact/close confinement with sylvatic animal reservoir • Social distancing • Hand hygiene • Avoid crowded places • Environmental disinfection • Face masks • Personal protective equipment for health care personnel	• Avoid contact with fresh- water that may be infested with schistosome parasites	• Avoid contact with faecal matter of wild animals and dogs • Hand hygiene after handling dogs	• Use of insect repellent, insecticides and bed nets	• Minimize contact with vector (i.e. tsetse flies)
**Main control strategies**	• Physical distancing (individual/school/workplace closures/border closures) • Case isolation • Contact tracing • Overall epidemic alert and response measures including risk communication	• Health education • Mass drug administration • Snail control strategies • Improved sanitation	• Health education • Limit the interactions between dogs and rodent populations • Prevent dogs from feeding on the carcasses of infected sheep • Control stray dog populations	• Early diagnosis and treatment, especially more efficacious drugs • Vector control • Control stray dog populations • Environmental management	• Active and passive case detection and treatment of confirmed cases • Vector control strategies • Management of the animal reservoir • Environmental management
**Challenges**	• Vaccine and drugs development • Improvement in animal health surveillance and further wildlife studies to increase knowledge on animal reservoir	• Vaccines • Improvement in animal health surveillance focused on cattle • Snail control	• Vaccine and new diagnostic methods • New drugs • Improved collaboration with animal health sector to improve surveillance (e.g. slaughter houses)	• Vaccine • New drugs • Improved reservoir and vector control methods • Improved control of stray dog populations where visceral leishmaniasis is most prevalent	• Timely diagnosis to avoid high out-of-pocket health expenditures • Vaccine • Development of new control methods • Surveillance strategies

*COVID-19* Coronavirus disease 2019, *SARS-CoV-2* Severe acute respiratory syndrome coronavirus 2

### Schistosomiasis

WHO calls for an integrated control strategy to face up to the challenge of 230 million people affected by this infection worldwide, mostly in sub-Saharan Africa [17]. Snail control is an important component of the overall control strategy, yet it has been found to pose considerable challenges. This is especially true in areas such as in the People’s Republic of China where schistosomiasis transmission persists as a zoonosis. Control and elimination in this country illustrates the complexity of some of the issues. The number of infected people was estimated to be 725,000 in 2005 [18]. Great progress has been achieved since then with infection rates dropping to < 0.001% in humans [19, 20]. In spite of continuous efforts by the Ministry of Health and partners, focal transmission persists in the lake region as a result of infection in cattle and resilience of the snail intermediate host posing a major risk for more than 30 million people in the endemic areas of the People’s Republic of China [21]. Unlike other schistosome species known to infect humans, *Schistosoma japonicum* is a true zoonotic organism, with a range of mammalian reservoirs, rendering control and elimination efforts difficult [22].

### Cystic echinococcosis

Cystic echinococcosis continues to represent a public health problem, affecting mostly disadvantaged people in Africa, the Americas and Asia [23]. These infections represent a burden adding to the already precarious situation of those affected, mainly poor farmers and herders throughout the endemic areas. Human incidence rates can be as high as 50 per 100,000 person-years, and prevalence levels as high as 5–10% in endemic regions. In South America, prevalence rates in slaughterhouses livestock can vary from 20 to 95%. For most countries in Africa only scarce data exist, if at all [24]. Nevertheless, it is conceivable that the combined burden posed by cystic echinococcosis and other infectious diseases affecting the very same population groups represents a major challenge to their health status and socioeconomic development. The paucity of effective tools to treat and prevent these infections represent a persistent obstacle illustrating the lack of interest by the relevant sectors in the development of new and efficacious drugs for many NTDs. It also highlights the lack of strategies, which could tap on the involvement of other sectors and key stakeholders behind a public health problem the determinants of which clearly reach well beyond the human health sector.

### Leishmaniasis

Comprised of multiple species of causative agents, leishmaniasis are among the top ten NTDs with more than 12 million infected people throughout the Americas, Africa, Asia and the Middle East [25]. This complex includes a particularly worrisome species for its morbidity, visceral leishmaniasis (VL), with 200,000 to 400,000 new cases per year worldwide, and an estimate of 20,000 to 40,000 deaths per year [26].

In 2017, 20,792 out of 22,145 new cases (94%) reported to WHO occurred in just seven countries; namely, Brazil, Ethiopia, India, Kenya, Somalia, South Sudan and Sudan [25, 27]. VL is prevalent in 12 countries in the Americas with 59,769 new cases reported from 2001 to 2017. Brazil accounts for 96% (57,582) of these cases [28]. The incidence rate of VL increased from 1990 to 2018 by 52.9% in this country alone with an even higher increase in children under the age of 1 year [28].

An additional challenge to effective control of this disease is canine visceral leishmaniasis (CVL), which constitutes a public health problem in some of the endemic countries. Cases of CVL are known to precede human cases of the disease, because dogs act as a reservoir. CVL plays an important role in the maintenance of transmission levels and the spread of the disease, particularly in urban areas [27, 29].

### Human African trypanosomiasis

Human African trypanosomiasis control programmes throughout Africa have been successful, recording a decrease of cases during the period 2001–2010 of 73.4% [30]. Yet, in spite of these impressive gains, delayed diagnosis of human African trypanosomiasis reported to have caused 93% of the affected households to experience an increase in financial expenditure (ranging from USD 60–170) in seeking treatment. To cover these out-of-pocket health expenditures, 81.5% of the affected families experienced difficulties in raising money for treatment resorting to various ways of raising it such as selling agricultural produce, seeking assistance from family and friends, selling/leasing of family assets, seeking credit and/or using personal savings [31].

It is to be expected that COVID-19 will greatly contribute to the impoverishment of these populations seriously jeopardizing decades’ long programmatic gains leading to a resurgence of human African trypanosomiasis.

### Challenges and recommendations: a case for a multi-sectoral strategy

We are currently facing one of the most serious public health crises in decades, the COVID-19 pandemic [32]. Most NTDs are subject to the impact of some of the very same human-related activities triggering other emerging and re-emerging diseases, such as COVID-19, SARS, bird flu and swine flu. The exact life cycle of COVID-19’s agent remains elusive to date [32]. The reason these epidemic-prone diseases attract more attention than the NTDs is that they can affect all, poor and rich, with extreme dire consequences in a short period of time. People can die within days of showing first symptoms. Just mentioning SARS or COVID-19 elicits a nervous response even from the financial sector. Economic losses have been consequently enormous. The key difference is NTDs affect primarily marginalized communities in low- and middle-income countries (LMICs), mainly rural and deprived urban communities. Economic losses, though significant, tend to be more selective in NTDs, affecting primarily the poor and medium income populations. While there is a frantic race among countries and pharmaceutical companies in COVID-19 for vaccines, drugs, diagnostics and protective gear, NTDs are not perceived as a lucrative market by the very same sectors hampering the development of diagnostics, drugs and vaccines.

COVID-19 could very well last for months, perhaps years. Unless we raise the profile of the NTDs, while the human population is highly sensitized to a public health crisis the likes of COVID-19, we might not get another opportunity to leverage on the raised awareness of emerging and re-emerging zoonotic diseases.

Health services and disease surveillance will need to undergo profound changes in order to find more effective ways of coping with emerging and re-emerging diseases in the future, zoonosis among these. The failure of most countries to respond adequately to COVID-19 makes a clear case for significant transformation of our systems. The NTD community would be well advised to jump on board. Integration of health and care services (e.g. group- and disease-specific models) [33] will need to be seriously considered in a post-COVID-19 world. It might also offer several potential venues to tackle morbidity in some NTDs. This issue deserves special attention, as it remains to be seen whether current integrated health care models could be extrapolated to NTDs in settings of LMICs.

An example of how non-health sector programme activities could be articulated with elements of NTD helminth control programme activities is described in a comprehensive analysis conducted in 2015. The analysis renders an interesting scenario, which should be further explored [9]. The One Health initiative is a movement to forge co-equal, all inclusive collaborations between physicians, osteopathic physicians, veterinarians, dentists, nurses and other scientific-health and environmentally related disciplines. However, it needs to ensure that other relevant sectors are included in the discussions of methodologies, definition of indicators and in the actual implementation of multicentre pilot studies to determine feasibility of multi-sectoral strategies. Given the uniqueness of each setting, meaning countries and continents, potential synergies with COVID-19 public health interventions (Table 2) and other sectors (Table 3) agendas would need to be tested and adapted to each particular social-ecological setting.

**Table 2:** Examples of where COVID-19 could synergize NTD efforts

Fields in mitigation	Opportunities for synergies with NTD control and elimination programmes
Overall surveillance capabilities strengthened in COVID-19 including surveillance sentinel sites	May improve NTD surveillance
Political stakeholders	Leverage for increased involvement in NTDs
Private stakeholders	Leverage for increased involvement in NTDs
Risk communications developed for epidemic prone diseases	May support greater community involvement and behavioural changes in NTDs
Media information dissemination	Piggybacking opportunity for NTDs
IT capabilities strengthened in COVID-19	Piggybacking opportunity for NTDS
Field logistics	Piggybacking opportunity for NTDs

*COVID-19* Coronavirus disease 2019, *NTDs* Neglected tropical diseases, *IT* Information Technology

**Table 3:** Examples of where other health and non-health sectors (private, public and not-for-profit) could synergize NTD efforts

Sector	Opportunities for synergies
Veterinary	• Disease surveillance (clinical and laboratory)
	• Field related logistics
	• One Health initiative
Policy	• Legislation
	• Assigning and/or increasing programme budgets
	• Streamlining financial flows to support effective supply chains
	• Implement strategies to promote economic development of affected populations (e.g. micro enterprises, revolving funds, etc.)
Economic	• Cost and feasibility studies
Marketing	• Tap on private sector expertise and adapt to use in health programmes
Communication	• Adapt private and public sector strategies
Pharmaceutical	• In conjunction with national academia and other organizations (e.g. Drugs for Neglected Diseases *initiative* (DND*i*)), support development of new drugs and diagnostics
Environmental	• Programmes targeting sustainable environment protection actions to prevent severe environmental degradation in NTD areas
Information Technology (IT)	• Integrated information systems
	• Access to inter-sectoral data bases
	• Geographic information systems
	• New surveillance systems
Transportation	• Private companies (e.g. beverage sector) support public health interventions
Malaria, tuberculosis and HIV/AIDS	• Include NTDs in a repackaging of public health interventions
	• Malaria, tuberculosis and HIV/AIDS benefit from integrated synergies
	• NTDs benefit from high media profile of the three big ones
Agriculture	• Encourage community development in NTD endemic areas by
	• enabling local produce and self-sufficiency;
	• organic farming;
	• assistance in placing produce in markets; and
	• One Health initiative
Education	• Access to rural and marginalized population groups
	• Sensitizing in health and environment topics
	• Incorporating health into educational messages and curricula
Anthropology	• Provide tools to enable integration of local cultural patterns into public health programmes
Psychology	• Integrated mental health programmes relying on ethical and culturally sensitive tools supporting vulnerable populations
Development	• Poverty alleviation programmes
	• Capacity building in income generating activities

## Conclusions

We believe that COVID-19 will exacerbate the NTD situation, as it will divert much needed financial and human resources. One might even anticipate a reversal in progress achieved in some of the NTD control and elimination efforts, similar to what is predicted for child and maternal health, malaria, tuberculosis and HIV/AIDS [34, 35]. Public health services are being stretched to their limits by COVID-19 and will continue to be so for some time to come. Other public health programmes will most likely suffer the consequences across the board. This will force the medical and the political establishments to make some extremely tough choices. We conjecture that COVID-19 will push a large proportion of the human population into poverty, even extreme poverty. To the NTD community, while we do face this rather dire scenario, it is imperative that we find alternative strategies if we want to retain the gains of NTD control and elimination programmes.

Future potential strategies will need to reconsider the determinants of health in NTDs in order to galvanize efforts and come up with comprehensive, well defined programmes that will set the stage for a multi-sectorial approach involving the medical, animal and human, developmental, environmental, agricultural, education, economy, anthropology, communications, public, private sectors, bilateral agencies and information technology sectors.

## Data Availability

Not applicable.

## Abbreviations

COVID-19: Coronavirus disease 2019
DALY: Disability-adjusted life year
GSK: Glaxo-Smith-Klein
HIV/AIDS: Human immunodeficiency virus/acquired immunodeficiency syndrome
IT: Information Technology
LMICs: Low- and middle-income countries
NTDs: Neglected tropical diseases
SARS: Severe acute respiratory syndrome
SARS-CoV-2: Severe acute respiratory syndrome coronavirus 2
WHO: World Health Organization
